# Severe respiratory disease caused by human respiratory syncytial virus impairs language learning during early infancy

**DOI:** 10.1038/s41598-020-79140-1

**Published:** 2020-12-21

**Authors:** Marcela Peña, Cristina Jara, Juan C. Flores, Rodrigo Hoyos-Bachiloglu, Carolina Iturriaga, Mariana Medina, Javier Carcey, Janyra Espinoza, Karen Bohmwald, Alexis M. Kalergis, Arturo Borzutzky

**Affiliations:** 1 Laboratorio de Neurociencias Cognitivas, Escuela de Psicología, Pontificia Universidad Católica de Chile, 7820436 Santiago, Chile; 2 División de Pediatría, Escuela de Medicina, Pontificia Universidad Católica de Chile, 8330077 Santiago, Chile; 3 Complejo Asistencial Dr. Sótero del Río, Servicio de Pediatría, 8207257 Santiago, Chile; 4 Departamento de Enfermedades Infecciosas e Inmunología Pediátrica, Pontificia Universidad Católica de Chile, 8330077 Santiago, Chile; 5 Millennium Institute On Immunology and Immunotherapy, Departamento de Genética Molecular y Microbiología, Facultad de Ciencias Biológicas, Pontificia Universidad Católica de Chile, 8330025 Santiago, Chile; 6 Departamento de Endocrinología, Facultad de Medicina, Pontificia Universidad Católica de Chile, 8330077 Santiago, Chile; 7 Departamento de Ciencias de la Salud, Facultad de Medicina, Pontificia Universidad Católica de Chile, 7820436 Santiago de Chile, Chile

**Keywords:** Cognitive neuroscience, Paediatrics

## Abstract

Human respiratory syncytial virus infection is a leading cause of pediatric morbidity and mortality. A previous murine study showed that during severe acute respiratory infections the virus invades the central nervous system, and that infected animals evolve with long-lasting learning difficulties associated with long-term potentiation impairment in their hippocampus. We hypothesized here that human infants who presented a severe episode of respiratory syncytial virus infection before 6 months of age would develop long-term learning difficulties. We measured the acquisition of the native phoneme repertoire during the first year, a milestone in early human development, comprising a reduction in the sensitivity to the irrelevant nonnative phonetic information and an increase in the sensitivity to the information relevant for the native one. We found that infants with a history of severe respiratory infection by the human respiratory syncytial virus presented poor distinction of native and nonnative phonetic contrasts at 6 months of age, and remained atypically sensitive to nonnative contrasts at 12 months, which associated with weak communicative abilities. Our results uncover previously unknown long-term language learning difficulties associated with a single episode of severe respiratory infection by the human respiratory syncytial virus, which could relate to memory impairments.

## Introduction

Human respiratory syncytial virus (hRSV) causes ~ 4.9% of the world’s episodes of acute lower respiratory tract infection in infants and children younger than five years^1–3^. The neurological complications of respiratory hRSV infections in human children appear in ~ 2% of cases^4–7^. The most frequent are central apnea, seizure, lethargy, swallowing alteration, strabismus, hypotonia, and encephalopathy (e.g. Refs.^4–7^). Occasionally, the neurological symptoms are associated with brain injury at the hippocampus, brainstem, cerebellum, and other regions of the cerebral cortex (e.g., Refs.^8–10^).

Although neurologic manifestations of severe hRSV infection have been exhaustively explored, the cognitive consequences associated with this clinical picture are highly unknown. Two recent studies with rodents infected with hRSV provided new insights about the issue. The first showed that during a severe acute respiratory infection by hRSV, the virus invades the rodent’s brain leading to difficulties in spatial learning and exploratory behaviors that were associated with long-term potentiation (LTP) impairment at the hippocampus. Importantly, the animals developed such impairments several months after the infection resolution, even when they did not show neurological symptoms during the acute infection or afterwards^11^. The second study identified possible pathogenic mechanisms for neurological damage associated with hRSV^12^ i.e. increased permeability of the blood–brain barrier and increased cytokine level and immune cell infiltration in the brain. Inspired by these animal studies, and by the scarcity of human studies about the cognitive consequences of viral respiratory infections during early infancy, we developed the current study.

**Figure 1:**
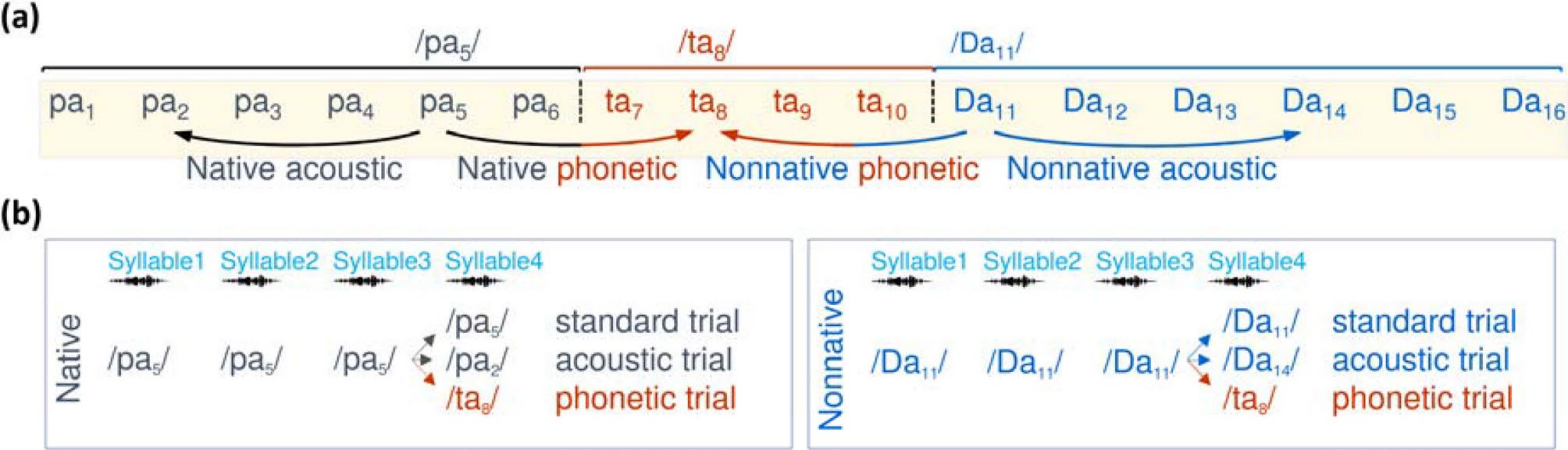
The native and nonnative phonetic distinction. In (**a**) we illustrate the synthetic consonant–vowel stimuli previously used to study categorical perception in English^1﻿3﻿^, Hindi^14﻿^, and Spanish-speakers^15^. This continuum comprises 16 steps (i.e. syllables) equidistant along the voiced place-of-articulation dimension from the bilabial /b/ to the dental /d/ and to the retroflex /D/, associated with the vowel /a/. Along the continuum, adult native English-speakers perceive two phonetic categories (Step1–Step6 as /ba/ and the following as /da/) while adult native Hindi-speakers perceive three (Step1–Step6 as /ba/, Step7–Step10 as /da/, and Step11–Step16 as retroflex /Da/). Adult Spanish-speakers also perceive two categories, however from Step1 to Step6 are perceived as /pa/ and the next as /ta/, due to a shorter voice onset time for these syllables in Spanish. In grey and orange, we indicate the syllables perceived as the native /pa/ and /ta/ respectively by Spanish-speaking adults, and in blue, those perceived as the nonnative /Da/ by Hindi-speaking adults. The phonetic boundaries are indicated by black vertical dotted lines. The arrow below the continuum indicates the stimulus we compared in this study to evaluate the response to phonetic and acoustic changes at the native and nonnative boundaries. In (**b**) we illustrate the structure of the trials. Each trial comprised the auditory presentation of four 275 ms long syllables, one every 600 ms separated by silence. The first 3 syllables were always identical, to induce habituation, while the 4th syllable remained the same in standard trials, changed to a syllable from a different phonetic category in phonetic trials or changed to a syllable of the same category in acoustic trials. Because any change between phonemes conveys a change at the acoustic and linguistic level, the subtraction of the brain response for standard and acoustic trials to the response for phonetic ones, at the native and nonnative phonetic boundaries, allowed us to quantify the purely linguistic component of the MMR.

We aimed to explore whether a severe acute episode of respiratory infection by hRSV, developed during the first months of life, associated with long-term learning difficulties, in infants who evolved without overt neurological manifestations, and remained asymptomatic after the infection recovered. We hypothesized that severe respiratory infections by hRSV would impair human learning during infancy. We estimated learning by measuring the building of the native phonemic repertoire, a milestone in language acquisition^13^. Infant’s behavioral^14^ and brain data^15,16^ have shown that before 9 months, infants distinguish native and nonnative phonetic contrasts, while by their first birthday they reduce their sensitivity to the nonnative ones, behaving as adults (Fig. 1a). Indeed, the amplitude of the brain response underpinning the detection of a change (henceforth MMR for mismatch response), is high at the native and nonnative phonetic boundaries at ~ 6 months, and it reduces at the nonnative boundary near ~ 12 months of age. Importantly, the amplitude of phonetic MMR correlates to later infants’ linguistic abilities. A reduced MMR amplitude for native contrasts before 12 months of age predicts reading difficulties^17–19^, and a reduced MMR amplitude for nonnative contrasts at 11 months predicts higher performance in word production at ~ 24 and ~ 30 months of age^20,21^.

We thus measured the phonetic MMR to estimate the learning of the native phonemic repertoire in four groups of infants. Two groups comprised infants with a history of a single severe hRSV respiratory acute episode without neurological manifestations before the 6 months of age, who evolved asymptomatic after the infection resolution, one evaluated at 6 months (V6), and the other at 12 months of age (V12). The other two groups of infants had no history of severe hRSV infection, evaluated as controls, one at 6 months (C6), and the other at 12 months of age (C12).

We predicted that the V6 group would elicit weaker MMR for the native and nonnative phonetic contrasts than the C6 group. We also predicted that the group V12 would present stronger MMR for the nonnative phonetic contrast and fewer communicative behavioral abilities than the group C12. Overall, we expected to provide new neurobehavioral data correlating the severe respiratory infections by hRSV to long-term language learning difficulties, which otherwise would remain hidden.

## Results

Considering our hypotheses at 6 and 12 months of age, we separately analyzed the data of 6- and 12-months-old infants. The groups did not differ in their demographic or clinical data at each age (Supplementary Table 1 and Supplementary Table 2).

### MMR analysis

**Figure 2:**
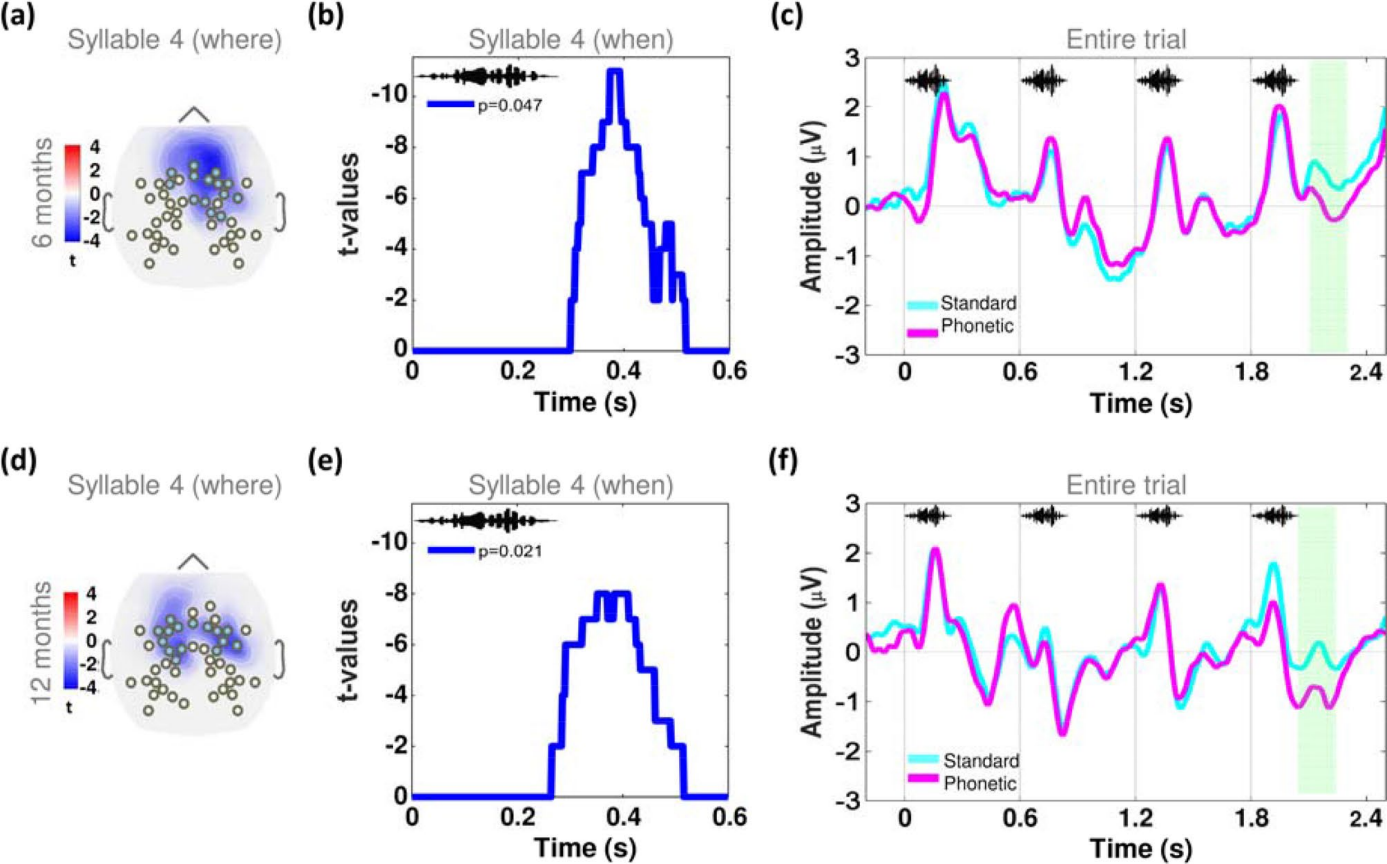
The spatiotemporal clusters for the analysis of the phonetic MMR was similar at 6 and 12 months of age. By applying the cluster-based permutation technique we identified where (i.e. the electrodes) and when (i.e. the time window) the brain response to the syllable 4 differed between the phonetic and standard trials, computed across all infants, regardless of the group (hRSV and control) and type of phonetic contrast (native and nonnative), at 6 and 12 months of age. The *t*-maps plotted in (**a**) and (**d**) show the group of the frontal electrodes where the standard and phonetic trials differed at syllable 4, at 6 and 12 months of age, respectively, and in (**b**) and (**e**) we depict the *t*-values indicating the time windows when those differences remained significant, at *p* < 0.05, FDR corrected. In (**c**) and (**f**) we plot the time course of the grand average of the voltage amplitude for the entire standard and phonetic trials, computed over the spatiotemporal cluster corresponding to age. The soundwave images indicate the occurrence of each one of the 4 syllables of the trials. The green rectangle highlights the time window when the brain response to the standard and phonetic trials differed.

We illustrate in Fig. 1b the experimental MMR protocol for the detection of change at the native and nonnative phonetic boundaries. We focused the MMR analysis on syllable 4. We applied a data-driven approach to identify spatiotemporal clusters (i.e. the electrodes and time windows), over which we had to test our group-related hypotheses without any a priori assumption. The cluster-based permutation analysis (see Methods) identified a ~ 200 ms long windows over frontal electrodes at 6 (Fig. 2a–c) and 12 months of age (Fig. 2d–f), when the brain's response for phonetic and standard trials differed across all infants, regardless of the group (hRSV and control) and phonetic contrast (native and nonnative). We then submitted to statistical comparisons the mean amplitude of the brain response over the spatiotemporal cluster corresponding to each age, computed in each infant of each group and for each phonetic contrast.

**Figure 3:**
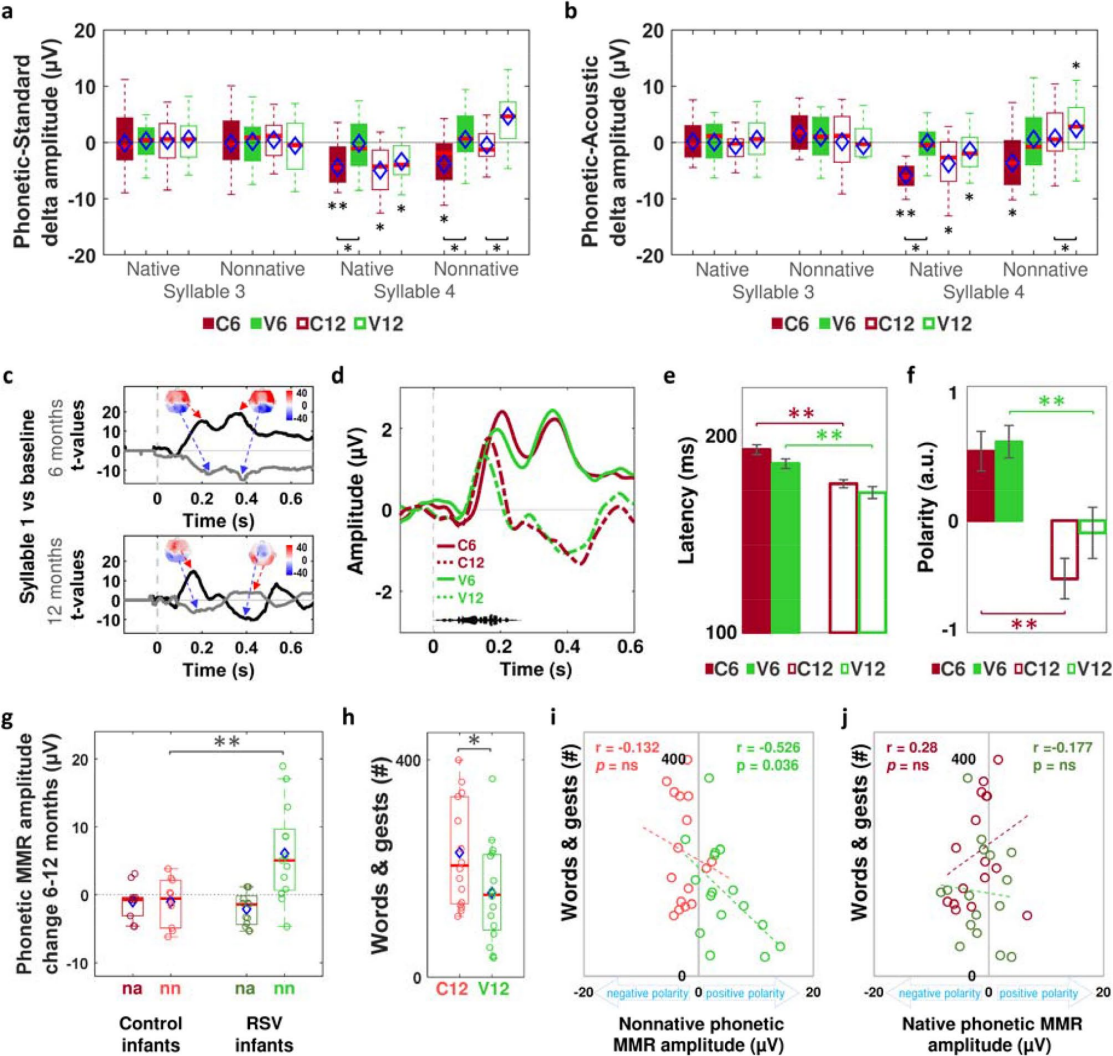
The V6 and V12 groups showed atypical phonetic MMR and fewer linguistic skills than the control groups. In (**a**) we depict the boxplots of the phonetic MMR for the native and nonnative contrasts, at syllable 3 and 4 per group. Red horizontal lines and blue diamonds correspond to the median and the mean of each group, respectively. Error bars indicate 95% confidence interval. The asterisks adjacent to the boxplots indicate significant differences from zero at *p* < 0.05, while the asterisks below the braces indicate significant differences between groups at the extremes of the braces. In (**b**) we plot the boxplots for the difference in amplitude between phonetic and acoustic trials, to extract the linguistic component of the phonetic MMR. In (**c**), we depict the *t*-map for the electrodes and *t*-values for the time window at the frontotemporal (black line) and parieto-occipital electrodes (grey line), over which the brain response for the syllable 1 differed from the silent baseline at 6 and 12 months of age. In (**d**) we plot the time course of the grand average for the syllable 1 per group and age, computed over the spatiotemporal windows at the frontal electrodes. We illustrate in (**e**), the mean of the first peak’s latency and in (**f**), the mean of the second peak polarity per group. We depict in (**g**) the difference in amplitude of the mean phonetic MMR between 6 and 12 months of age, in a subsample of infants evaluated at both ages, nn = nonnative; na = native. In (**h**) we depict the sum of words and gests understood and used by infants of the C12 and V12 groups, reported by their parents. In (**i**) and (**j**) we plot the correlations of the mean sum of words and gests handled by the infants against the phonetic MMR amplitude for the nonnative and native phonetic MMR, respectively, in C12 (red circles) and V12 groups (green circles). Pearson’s correlation coefficient and *p*-value are indicated for each plot.

We started verifying that the differences in amplitude of the brain response for standard and phonetic trials occurred only at syllable 4, and not at syllable 3, when signals should not differ. We first computed the difference in amplitude between phonetic and standard trials and submitted this phonetic-standard delta amplitude to a comparison against zero, where zero indicated no difference. We found that the phonetic-standard delta amplitude was significantly different from zero only at syllable 4 (Fig. 3a) in group C6 for the native and nonnative phonetic contrast (*t*_(20)_ = −  3.470, *p* = 0.002, Cohen’ d = −  0.757, and *t*_(20)_ =  −  2.249, *p* = 0.022, Cohen’ d = −  0.491, respectively), in group C12 for the native contrast only (*t*_(22)_ =  −  2.552, *p* = 0.017, Cohen’ d = −  0.532), and in group V12 for the native and nonnative contrasts (*t*_(19)_ =  −  2.708, *p* = 0.015, Cohen’ d = −  0.605, and *t*_(19)_ = 3.763, *p* = 0.002, Cohen’ d = 0.841, respectively). As predicted, the phonetic MMR in the V6 group did not differ from zero for the native and nonnative contrasts at syllable 3 and 4 (*p* > 0.309), which we interpreted as difficulties distinguishing native and nonnative phonetic contrasts in this group.

After demonstrating the existence of differences in the phonetic-standard delta amplitude only at syllable 4 in most groups, we henceforth called this delta amplitude “phonetic MMR”. We then evaluated the role of the group factor in the phonetic MMR by submitting its amplitude to two separated repeated measures ANOVA, one per age, with type of phonetic contrast (native vs nonnative) as a within-subjects factor, and group (V6 vs C6 at 6 months of age, or V12 vs C12 at 12 months of age) as a between-subjects factor. As predicted, at 6 months of age we found an effect of group (*F*_(1,44)_ = 9.793, *p* = 0.003, ηp2 = 0.176), because the phonetic MMR was more negative in C6 than in V6 group for the native (*F*_(1,44)_ = 6.420, *p* = 0.015, ηp2 = 0.122) and nonnative contrasts (*F*_(1,44)_ = 5.975, *p* = 0.018, ηp2 = 0.115), without a group x type phonetic contrast interaction. At 12 months, we found a significant group x type phonetic contrast interaction (*F*_(1,41)_ = 4.420, P = 0.042, ηp2 = 0.097), because the phonetic MMR amplitude was significantly greater in group V12 than in group C12 for the nonnative contrast only (*F*_(1,41)_ = 8.075, *p* = 0.007, ηp2 = 0.165) (Fig. 3a). Unexpectedly, the polarity of the phonetic MMR for the nonnative contrast was positive in 17 out of 20 infants of group V12, while only 9 out of 23 infants of group C12 showed such characteristic (Fisher's Exact Test, *p* = 0.004). The positive polarity of the MMR has been reported in infants and children exposed to phonetic distinctions hard to detect^21^, infants with poor linguistic stimulation^22^, children with specific language impairment^23^ or family history of dyslexia^24^, and in immature infants before 5 months of age^25,26^. Because in our study the infants from C12 and V12 groups exhibited similar negative MMR for native contrast, none reported a family history of language difficulties and were very similar in age, gender, socioeconomic and cultural status, we speculated that the MMR’s positive polarity for nonnative phonetic contrasts in group V12 was related to greater difficulties to process the unfamiliar features of the foreign contrast, to which they remained atypically sensitive despite its irrelevance for their native language.

So far, our EEG results supported our predictions about language difficulties in hRSV groups, showing poor phoneme distinction in group V6 and robust response to the nonnative contrast in group V12. We then proceeded to demonstrate the linguistic nature of these MMR’s atypicalities.

We first excluded the possibility that group differences in phonetic MMR arose from differences in processing the acoustic properties of the phonemes. We computed the phonetic-acoustic delta amplitude by subtracting from the phonetic MMR amplitude, the amplitude of the MMR caused by the detection of an acoustic change between two exemplars of the same phonetic category (i.e. /pa_5_/ vs /pa_2_/ and /Da_11_/ vs /Da_14_/ in Fig. 1a). At 6 months, we found an effect of group (*F*_(1,44)_ = 12.501, *p* = 0.001, ηp2 = 0.214) because the phonetic-acoustic delta amplitude was greater in group C6 than in group V6 for the native contrast (*F*_(1,44)_ = 11.558, *p* = 0.001, ηp2 = 0.201), without a group x type of phonetic contrast significant interaction (*p* = 0.419). Although the phonetic-acoustic delta amplitude for the nonnative contrast was greater in group C6 than in group V6, this difference did not reach statistical significance (*p* = 0.051). Additionally, at 12 months we found a group x type of phonetic contrast significant interaction (*F*_(1,41)_ = 8.071, *p* = 0.007, ηp2 = 0.164) because, only at the nonnative phonetic contrast, the phonetic-acoustic delta amplitude was significantly more positive in the V12 than in the C12 group, (*F*_(1,41)_ = 16.585, *p* < 0.001, ηp2 = 0.288) (Fig. 3b).

By analyzing the brain response to the syllable 1, we also ruled out the possibility that hRSV infections had affected the processing of any speech sound in groups V6 and V12. Similar to the MMR analysis, we first identified the spatiotemporal windows over which response to both syllables 1 (i.e. /pa/ and /Da/ for the native and non-native trials respectively) maximally differed from the silent baseline across all infants, regardless of the group and type of trial. We found a response with two maximal components (at *p* < 0.001): the first peaking at ~ 186 ms and ~ 164 ms, in 6 and 12-month-old children, respectively, and the second peaking at ~ 440 ms and ~ 464 ms, in 6 and 12-month-old children, respectively (Fig. 3c). Confirming previous developmental ERP data^27,28^, the first peak had positive polarity over frontotemporal electrodes, and negative over the occipital ones at both ages, while the second peak maintained the same polarity at 6 months, but inverted it in 12-month-old infants (Fig. 3d). We then submitted the mean amplitude and mean latency of the peaks of each infant over the frontotemporal electrodes to two separated one-way ANOVA, one per age, with group (V6 vs. C6 at 6 months of age, or V12 vs. C12 at 12 months of age) as the between-subjects factor. We did not find group differences in any comparison (*p* > 0.411). Moreover, both hRSV and control groups exhibited similar age-related changes in syllable 1′s peaks, with a first peak latency 17.443 ms longer in group C6 than in group C12 (*t*_(43)_ = 5.396, *p* < 0.001, Cohen’s d = 1.61), and 14.756 ms longer in group V6 than in group V12 (*t*_(44)_ = 3.787, *p* = 0.001, Cohen’s d = 1.16) (Fig. 3e). A second peak’s polarity resulted more positive in group C6 than in group C12 (*t*_(43)_ = 4.603, *p* < 0.001, Cohen’s d = 1.39), and in group V6 than in group V12 (*t*_(44)_ = 2.927, *p* = 0.003, Cohen’s d = 0.92) (Fig. 3f). The lack of differences in the polarity of the brain response to syllable 1 between hRSV and control groups, at 6 and 12 months, contrasted with the differences we observed in the phonetic MMR’s polarity at 12 months, supporting the idea that the groups did not differ in the processing of phonemes per se, but in distinguishing them. An additional intra-subject analysis supported this interpretation: when we compared the subsamples of infants who were evaluated at 6 and 12 months of age in the control (n = 10) and hRSV (n = 13) groups, we replicated the results found for the full sample, showing that only in the hRSV groups the phonetic MMR for the nonnative contrast became more positive at 12 months of age (Mann Whitney *U* = 20.5, *p* = 0.005, critical U value at p < 0.05 = 33) (Fig. 3g).

Together, the brain results in the hRSV groups unveiled two atypical neural signatures previously associated with language difficulties^17–21^ i.e. low sensitivity distinguishing native and nonnative phonemes at 6 months, and high sensitivity distinguishing the nonnative phonetic contrast at 12 months. We proceeded then to evaluate the infants’ linguistic behaviors at ~ 12 months of age.

### Communicative abilities

We compared the linguistic capacities in thirty-two 12-month-old children (16 from group C12 and 16 from group V12), reported by the infants’ parents applying the MacArthur Communicative Developmental Inventories^29^ (Spanish adaptation)^30^. We found that the sum of first phrases, words, and gests that the infants understood, said, or used was significantly greater in group C12 than in group V12 (*t*_(30)_ = 2.278, *p* = 0.030, Cohen’s d = 0.83). The behavioral results indicated thus, that, from the caregiver’s perspective, near their first year of age the infants who suffered a severe hRSV respiratory infection exhibited less communicative behaviors than those without a history of a severe episode of hRSV respiratory infection (Fig. 3h).

### Neurobehavioral relationships

In 12-month-olds, we found that, only in group V12, the more positive the MMR for the nonnative contrast, the smaller the communicative abilities (*p* = 0.036) (Fig. 3i,j). This result agreed with previous neurobehavioral data^20,21^.

### Multivariate analysis and multiple regression analysis

The multivariate analysis of variance at 6 and 12 months ruled out the possibility that maternal education, maternal age, infant’s age, or infant’s gender predicted the hRSV and control groups. In contrast, when we added to the multivariate model, the phonetic MMR amplitude for native and non-native phoneme (or the Communicative abilities at 12 months), the model correctly predicted the groups at 6 and 12 months of age (see Table S3 and Table S4, respectively). Additionally, the multiple regression analysis confirmed the multivariate analysis by showing that the factor “group” explained the differences in the native (Table S5) and nonnative (Table S6) phonetic MMR amplitude at 6 months of age. Without significant effects of maternal education, maternal age, infant’s age, or infant’s gender (see Figure S3, panels a and b). At 12 months, the variance in the nonnative phonetic MMR amplitude was significantly explained by the factor “group” but also by the factor "maternal education" (Table S7a). However, the effect of maternal education was mainly due to one participant from the group V12 (see Fig. 3, panel d), who had a mother reporting education at level 1 (full primary education). When we removed from the regression this case (Table S7b), the factor "maternal education" did not explain the variance in the nonnative phonetic MMR amplitude anymore, while the factor "group" did it. Additionally, at 12 months, the variance in the communicative abilities was explained only by the factor “group” at 12 months of age (Table S8a and Table S8b). Finally, the multiple regression analysis restricted to the group V6 (Table S9) and group V12 (Table S10), including the "severity" of the infection as a factor, did not explain the variance on the brain and behavioral responses we found that differed between hRSV and control groups.

## Discussion

We showed that a severe hRSV respiratory infection suffered before 6 months of age was associated with long-term impairments in phonemes distinction at 6 months, and difficulties to learn the native phoneme repertoire and poor communicative abilities at 12 months of age. The impairments appeared even though the infants from hRSV and control groups did not significantly differ in infants' age, infants' sex, maternal education, maternal age, and socioeconomic level, and, beyond the history of severe hRSV infection, had a similar developmental history. Similar to the data reported in previous murine studies, we observed learning difficulties several months after the infection recovery, and in infants who did not develop neurological manifestations during the acute infection and afterward, and evolved without severe health problems.

Developmental literature has demonstrated that brain networks can make phonemic discriminations as early as 27 weeks of postconceptional age in healthy preterms^31^. However, the early steps of language acquisition are influenced by biological constraints^15,32^, and the stabilization of the linguistic representations in response to environmental stimulation depends on cortical circuitry reshaping to advance into an accurate perceptual tuning to the native language environment^16^. Our results indicate that a severe hRSV respiratory infection at early age can alter these processes. This alteration becomes relevant because previous studies have shown that the combination of difficulties in speech comprehension and production before 18 months of age associate with language impairment at older ages in up to 40% of cases^33^. Of course, further longitudinal studies are necessary to elucidate whether the early language difficulties we report here associate with language and communicative difficulties at preschool, school, and older ages.

Critically, the atypical brain responses for the phoneme distinction we report here cannot be explained by impairments in the auditory function or in an atypical development to process phonemes, potentially caused by the hRSV infection. Indeed, the perception of phonemes at syllable 1 as well as the expected maturational changes of the brain’s response to this syllable were similar in hRSV and control groups.

Further studies are necessary to measure the long-term effects of other factors reported as relevant for early cognitive development such as hospitalization, mother’s education, and mother’s age. Indeed, the lack to prove any effect of maternal education and maternal age on our results could be due to the small size of our samples.

Although more studies are necessary to identify the mechanisms underpinning the linguistic learning impairments we found, we propose that severe hRSV infections impair, at least transiently, the typical development of memory-related brain networks. The MMR has been conceived as a robust biological marker for both, sensory memory trace, since it appears by the detection of a change^34^, and recent memory-based predictions, given that it also displays when participants detect the omission of an expected stimuli^35^. To some extent, such memory impairment mirrors the one observed in rodents.

Albeit our results were significant, they were correlational. We did not demonstrate the direct compromise of the central nervous system (CNS) by hRSV, because it is not feasible to apply invasive study techniques of CNS involvement (e.g. lumbar puncture) to infants who evolved without neurological manifestations. We thus can only speculate about how the hRSV could affect cognition.

First, the increase in the viral load at the upper respiratory tract during any respiratory infection, regardless of the severity of the clinical picture, provides the pathogens with the opportunity to spread into the CNS via the olfactory bulb or be carried to the CNS by infected white blood cells. Moreover, previous studies, with infants who evolved with neurological symptoms, reported that the hRSV can harm the brain function by direct invasion (e.g. Ref.^36,37^) or through the production of intracerebral pro-inflammatory cytokines (e.g. Refs.^38,39^). Further studies are necessary to evaluate whether the atypical brain response and communicative impairments we reported here may correspond to such types of subclinical alterations of the CNS.

Another question to answer is whether the cognitive difficulties associated with hRSV infection depend on the hRSV genotypes. hRSV strains are classified in two major groups A and B and viruses from both groups usually co-circulate in a given season. Strains within each group can be classified in many genotypes. A number of previous studies showed no association between groups or genotypes with and the severity of the clinical picture (e.g. ref. ^40–42^). Alternatively, other studies link the sensitivity and severity of the clinical picture with hRSV genotypes^43,44^. For instance, a recent 12-years longitudinal study^45^ reported that the genotype RSV-A NA1 associates with more severe bronchiolitis than genotypes ON1 and BA, while the genotypes ON1 and BA would cause less severe bronchiolitis and would affect mainly infants who possibly have a genetic predisposition to asthma and atopy. Future studies are necessary to test the eventual role of the hRSV groups and genotypes on the cognitive abnormalities we observed in hRSV groups.

In perspective, we expect that our data may contribute to generate new hypothesis on: (a) the cognitive adaptations that young infants develop after acute viral respiratory infections; (b) the design of sensitive protocols of early detection of linguistic difficulties, which are highly under-reported; and (c) the development of preventive initiatives, such as hRSV vaccines. The last point is particularly promising, since, in rodents, the administration of a recombinant BCG-N-hRSV vaccine prevent the long-term neurocognitive impairments secondary to hRSV infection11. We also expect to encourage further explorations about the linguistic consequences of pandemic viral respiratory infections, given that language is crucial shaping cognition from very early on^46^.

## Methods

### Participants and approvals

We evaluated 99 infants. Ten were excluded from the study (4 V6, 1 V12, and 3 C6 and 2 C12) because they did not complete the experimental protocol or contributed with less than 14 non-artifacted EEG trials of each type (i.e. standard, phonetic and acoustic). We thus report on 25 V6 (13 male), 20 V12 (12 male), 21 C6 (12 male), and 23 C12 (12 male). The sample size of each group was similar and even larger than others previously reported for MMR study in young infants^15,21,47^. Thirteen infants from the group V6 and 10 from the group C6 were also evaluated at 12 months of age.

All infants belonged to a middle-low socioeconomic class, monolingual Spanish-speaking environment, and presented a history of normal physical, psychomotor, and sensorial development at the test time (Supplementary Table 1 and Supplementary Table 2). None have a history of specific language impairment or family dyslexia.

We confirmed the hRSV etiology for the infection by using indirect immunofluorescence of nasopharyngeal swabs. We excluded from the study the hRSV patients who required invasive mechanical ventilation to avoid confounding effects on developmental milestones associated with the use of sedative drugs and muscle relaxants. We purposefully excluded from the study infants with chronic conditions that predispose them to severe hRSV disease, including extreme prematurity, bronchopulmonary dysplasia, congenital heart disease, immunodeficiency or immunosuppression. We also excluded patients and healthy controls with any disease that per se causes or predisposes to developmental alterations, including neonatal asphyxia, epilepsy, chromosomal disorders, and inborn errors of metabolism. The oxygen saturation of the infants from the groups hRSV maintained above 90% from the admission to the discharge. During hospitalization, the infants received non-invasive ventilation support when necessary. We prospectively recruited the hRSV patients during their stay at the Hospital and the infants from the control groups during routine well-child ambulatory visits. Mothers or other caregivers were allowed to remain hospitalized with their infants, to avoid the negative impact on the further development of the separation of the infants with their caregivers during hospitalizations.

Our study received the approval of the Institutional Ethics Committee of the Pontificia Universidad Católica de Chile. All methods followed the relevant guidelines and regulations of the mentioned committee. The infants’ parents signed a written consent form to allow their infants to participate in the study.

### Procedure for EEG recordings

We tested infants in a soundproof Faraday booth. The infant sat on the parent’s lap and the parent listened to music through earphones to mask the speech stimuli during testing. Infants heard 30 trials in each of six experimental conditions, that arose from the combinations of three types of trial (i.e., standard, acoustic, and phonetic) and two types of phoneme contrast (i.e., native and nonnative). In each trial we played 4 syllables (275 ms long each), one every 600 ms separated by silence. The inter-trial intervals randomly varied between 3500 and 4000 ms. Syllables were delivered by loudspeakers at 60 dB SPL. To reduce body movements and control the infant´s attention, 1000–1500 ms before the trial onset we presented a babbling sound associated with an infant face, randomly selected from a pull of 40 faces, displayed at the center of a screen, placed in front of the infant. The infant face's image remained on the screen until the trial ended. We played video breaks during trials every time we needed to reset the infants’ attention. Infants could play with a small toy during testing.

### EEG data acquisition, processing, and statistical analysis

We continuously recorded the scalp voltages by using a 64-channels EEG system (EGI, Inc., Eugene, OR), digitized at a sampling rate of 500 Hz. The maximal impedance was 40 kΩ. The EEG signal was first filtered (bandpass filter = 0.5–20 Hz) and then segmented into epochs lasting 3500 ms, including 500 ms preceding the first syllable of the trial. Epochs containing electrodes with voltage fluctuations exceeding 150 µV or transients exceeding 100 µV were rejected. Non-rejected epochs were averaged, baseline-corrected (across the 200 ms before the onset of the first, third, and fourth syllable, depending on the analyzed syllable), and transformed into an average reference. Each infant contributed with 14 to 30 non-rejected trials per experimental condition. For the described procedures, we used EEGLAB^48^, an open-source Matlab toolbox for EEG analysis. For statistical analysis, we applied a data-driven approach to identify spatial (i.e., electrodes) and temporal (i.e., time windows) clusters when comparing the brain response for different experimental conditions across groups. For MMR analysis, we applied a cluster-based permutation analysis (with 1000 iterations, threshold of *p* = 0.05, FDR corrected), which is an unbiased nonparametric procedure developed by Fieldtrip^49^ and implemented in Brainstorm^50^, both open-source Matlab toolboxes for EEG analysis. For the first analysis of the syllable 1, we applied a one-sample t-test comparing brain activity during stimulation against the brain activity along the 200 ms prestimulus baseline. After identifying the spatiotemporal windows, we computed in each infant of each group, and for each phonetic contrast, the mean MMR amplitude over each cluster corresponding to each age. These averages were submitted to a one-sample t-test against zero change (2 tails, alpha = 0.05) and to repeated measure ANOVAs to explore differences related to the factor "group". The *p*-values were corrected for multiple comparisons to adjust the family-wise error rate for each comparison, using the Bonferroni correction in t-test and Greenhouse–Geisser correction in repeated measure ANOVAs. For the intra-subject comparison, we applied the Mann Whitney rank test, suitable to analyze small samples with unequal variance.

### Communicative skills

Communicative skills. We estimated the infant linguistic skills by using a Spanish adaptation of the MacArthur Communicative Development Inventories, which have a high test–retest reliability in different populations, including the Latin Spanish-speaking one^30^. The MacArthur Communicative Development Inventories^29^ asks caregivers to report about the infant's understanding of first phrases and the infant's comprehension and production of vocabulary and gestures. For each item, the caregiver must indicate if the infant just understood, understood-and-said, or used it. We analyzed the sum of all items handled by each infant at about 12 months of age.

## Supplementary information

Supplementary Information 1.
