# Supplementary material for: Severe respiratory disease caused by human respiratory syncytial virus impairs language learning during early infancy
Source: Sci Rep. 2020 Dec 21;10:22356. doi: 10.1038/s41598-020-79140-1 (PMC7752900; doi:10.1038/s41598-020-79140-1)
Supplement: Supplementary file 1 — Supplementary Information 1. [file 41598_2020_79140_MOESM1_ESM.docx]

Supplementary information (revised2)

**Severe respiratory disease caused by human respiratory syncytial virus impairs language learning during early infancy.**

Marcela Peña, Cristina Jara, Juan C. Flores, Rodrigo Hoyos-Bachiloglu, Carolina Iturriaga, Mariana Medina, Javier Carcey, Karen Bohmwald, Alexis M. Kalergis & Arturo Borzutzky.

| **Table S1.** Demographic & pediatrics data per group. | | | | |
| --- | --- | --- | --- | --- |
|  | Group | Median | Min | Max |
| Apgar 5 min | C6 | 9 | 8 | 10 |
|  | V6 | 9 | 8 | 10 |
|  | C12 | 10 | 8 | 10 |
|  | V12 | 9 | 9 | 10 |
| Gestational age (weeks) | C6 | 39 | 36 | 41 |
|  | V6 | 39 | 36 | 41 |
|  | C12 | 39 | 36 | 41 |
|  | V12 | 38.5 | 36 | 41 |
| Birth weight (gr) | C6 | 3263 | 2650 | 4500 |
|  | V6 | 3240 | 2090 | 4120 |
|  | C12 | 3500 | 2820 | 4390 |
|  | V12 | 3605 | 2900 | 4120 |
| Height (cm) | C6 | 49.8 | 41 | 54 |
|  | V6 | 50 | 45 | 60 |
|  | C12 | 49 | 41 | 99 |
|  | V12 | 51.5 | 49 | 65 |
| Test age (months) | C6 | 5.1 | 3.3 | 7.2 |
|  | V6 | 4.9 | 2.1 | 6.9 |
|  | C12 | 12.9 | 12 | 14.9 |
|  | V12 | 13.1 | 12 | 14.7 |
| Maternal age (years) | C6 | 29 | 19 | 35 |
|  | V6 | 24 | 17 | 42 |
|  | C12 | 28 | 19 | 35 |
|  | V12 | 27 | 17 | 42 |
| Maternal education (level) | C6 | 2 | 2 | 3 |
|  | V6 | 2 | 1 | 3 |
|  | C12 | 2 | 2 | 3 |
|  | V12 | 2 | 1 | 3 |
| Maternal education level 1: full primary; 2: full secondary; 3: college or higher. Differences between control and hRSV groups were not significant (*P* > 0.35 for each comparison) | | | | |

| **Table S2. Clinical data for hRSV groups.** | | | | |  | |
| --- | --- | --- | --- | --- | --- | --- |
|  |  | **Group** | **Median** | **Min** | | **Max** |
| **Clinical syndrome** | Bronchiolitis | V6 | 21 |  | |  |
|  |  | V12 | 16 |  | |  |
|  | Pneumonia | V6 | 4 |  | |  |
|  |  | V12 | 4 |  | |  |
| **Acute episode** | Age at the infection (months) | V6 | 2.2 | 1.0 | | 5.5 |
| **Therapy** |  | V12 | 2.5 | 1.0 | | 5.5 |
|  | Months after infection resolution | V6 | 2.4 | 1.0 | | 5.0 |
|  |  | V12 | 10.8 | 6.8 | | 13.5 |
|  | Oxygen therapy (days) | V6 | 9.0 | 7.0 | | 16.0 |
|  |  | V12 | 9.0 | 7.0 | | 16.0 |
|  | Hospitalization (days) | V6 | 10.0 | 8.0 | | 18.0 |
|  |  | V12 | 10.0 | 8.0 | | 18.0 |
|  | Non-invasive mechanical ventilation (days) | V6 | 4.5 | 1.0 | | 9.0 |
|  |  | V12 | 4.0 | 2.0 | | 9.0 |
|  | Severity score (a.u.) | V6 | 8.3 | 5.3 | | 13.0 |
|  |  | V12 | 8.5 | 6.0 | | 12.7 |
| a.u.: arbitrary units. Differences between hRSV groups were not significant (*P* > .42 for each comparison) | | | | | | |


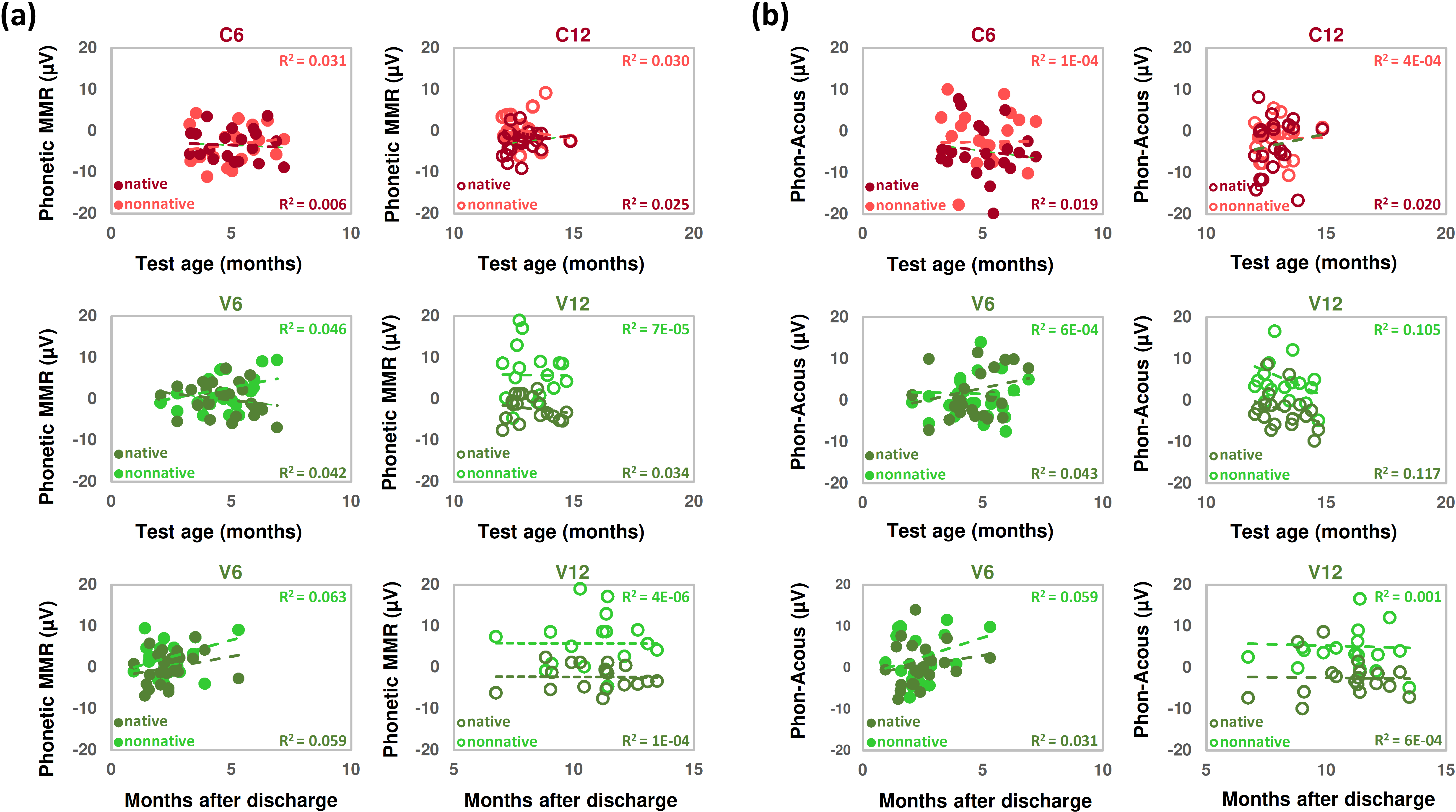


**Figure S1**. **The brain response to the phonetic changes did not correlate with the test age in all infants neither with the duration of the period between the infection resolution and the test in hRSV groups**. In (**a**), we plot the correlations between the phonetic MMR amplitude, for the native and nonnative contrasts, against the test age in all infants, and against the months after discharge at home in hRSV groups. In (**b**), we depict the correlations between the test age and months after discharge against the difference in amplitude of the brain response for phonetic and acoustic trials, to estimate the linguistic component of the phonetic MMR. We write the R^2^ of each correlation in each plot. We did not found any significant correlation at *p* < 0.05.


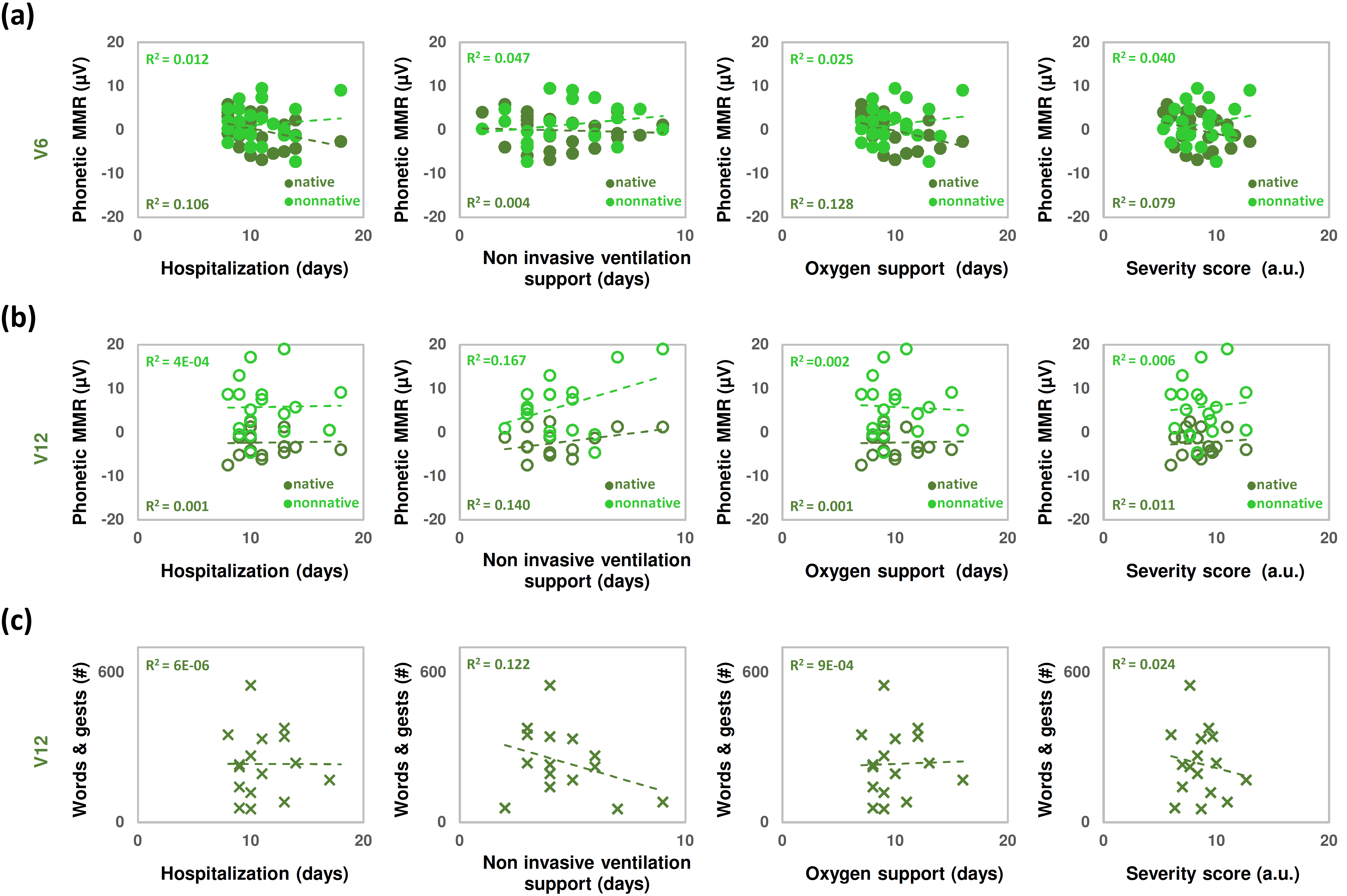


**Figure S2**. **The phonetic MMR amplitude at 6 and 12 months of age, and the linguistic abilities at 12 months of age did not correlate with the severity of the infection in hRSV** **groups.** In (**a**) and (**b**), we plot the bi-variate correlations between the phonetic MMR amplitude against the days of hospitalization, the days of non-invasive ventilation support, days of oxygen support and severity score, computed as the average of the previous 3 values, for the infants of the V6 and V12 groups respectively. In (**c**), we depict the bi-variate correlations between the sum of the words and gests that the infant handle, reported by the infants’ parents against the days of hospitalization, the days of non-invasive ventilation support, days of oxygen support and severity score for the infants of the V12 groups. We write the R^2^ of each correlation in each plot. We did not found any significant correlation at *p* < 0.05.

| **Table S3**. The table shows the main statistical indexes for the multivariate analysis of variance (MANOVA) at 6 months of age, in (a) including four demographic variables in the model, and in (b) adding the brain variables. Only the brain variables successfully predicted the V6 and C6 groups. | | | | | |
| --- | --- | --- | --- | --- | --- |
| **MANOVA** | **Df** | **Wilks** | **F-Statistic** | **p-value** | **Multiple Adjusted R-squared** |
| (a)  Maternal education *  Maternal age *  Infant Gender *  Infant Age | 4,34 | 0.911 | 0.771 | 0.561 | -0.006 |
| b)  Maternal education *  Maternal age *  Infant Gender *  InfantAge *  Native Phonetic MMR amplitude *  NonNative Phonetic MMR amplitude | 6,32 | 0.453 | 5.83 | < 0.001 | 0.211 |
|  |  |  |  |  |  |
| **UNIVARIATE ANALYSIS** | **Df** | **F-Statistic** | **p-value** |  |  |
| Maternal education | 1,37 | 1.398 | 0.245 |  |  |
| Maternal age | 1,37 | 0.078 | 0.781 |  |  |
| Infant Gender | 1,37 | 1.368 | 0.25 |  |  |
| Infant Age | 1,37 | 0.771 | 0.386 |  |  |
| Native Phonetic MMR amplitude | 1,37 | 19.825 | < 0.001 |  |  |
| NonNative Phonetic MMR amplitude | 1,37 | 10.358 | 0.002 |  |  |
| * 8 observations deleted due to missingness. Mothers did not provide their age or educational level. | | | | | |

| **Table S4**. The table shows the main statistical indexes for the multivariate analysis of variance (MANOVA) at 12 months of age, in (a) including four demographic variables in the model, and in (b) adding the brain variables and Communicative Development Inventories score (also called CDI). Only the brain variables and CDI score successfully predicted the V12 and C12 groups. | | | | | |
| --- | --- | --- | --- | --- | --- |
| **MANOVA** | **Df** | **Wilks** | **F-Statistic** | **p-value** | **Multiple Adjusted R-squared** |
| (a)  Maternal education *  Maternal age *  Infant Gender *  Infant Age | 4,34 | 0.897 | 0.86 | 0.498 | 0.032 |
| b)  Maternal education *  Maternal age *  Infant Gender *  Infant Age *  Native Phonetic MMR amplitude * NonNative Phonetic MMR amplitude | 6,34 | 0.459 | 5.5 | < 0.001 | 0.443 |
|  |  |  |  |  |  |
| **UNIVARIATE ANALYSIS** | **Df** | **F-Statistic** | **p-value** |  |  |
| Maternal education | 1,36 | 0.531 | 0.471 |  |  |
| Maternal age | 1,36 | 0.216 | 0.645 |  |  |
| Infant Gender | 1,36 | 0.66 | 0.422 |  |  |
| Infant Age | 1,36 | 2.109 | 0.156 |  |  |
| Native Phonetic MMR amplitude | 1,36 | 0.561 | 0.459 |  |  |
| NonNative Phonetic MMR amplitude | 1,36 | 28.007 | < 0.001 |  |  |
| CDI score | 1,24 | 6. 914 | 0.014 |  |  |
| * 6 observations deleted due to missingness of maternal age or education.  * 14 observations deleted due to missingness in CDI data. | | | | | |

| **Table S5.** Regression models for native phonetic contrast in 6-months-old infants. | | | | |  | **Table S6.** Regression models for nonnative phonetic contrast in 6-months-old infants. | | | | |
| --- | --- | --- | --- | --- | --- | --- | --- | --- | --- | --- |
| **MODEL INFO:** | | | | |  | **MODEL INFO:** | | | | |
| Observations: 38 (8 missing obs. deleted) | | | | |  | Observations: 38 (8 missing obs. deleted) | | | | |
| Dependent Variable: native Phonetic MMR amplitude | | | | |  | Dependent Variable: nonnative Phonetic MMR amplitude | | | | |
| Type: OLS linear regression | | | | |  | Type: OLS linear regression | | | | |
|  | | | | |  |  | | | | |
| **MODEL FIT: 1** | | | | |  | **MODEL FIT: 1** | | | | |
| F(6,31) = 3.31, p = 0.01** | | | | |  | F(6,31) = 5.11, p = 0.00*** | | | | |
| R² = 0.39 | | | | |  | R² = 0.50 | | | | |
| Adj. R² = 0.27 | | | | |  | Adj. R² = 0.40 | | | | |
|  | | | | |  |  | | | | |
| Standard errors: OLS | | | | |  | Standard errors: OLS | | | | |
|  | Est. | S.E. | t | p |  |  | Est. | S.E. | t | p |
| (Intercept) | -0.82 | 6.59 | -0.12 | 0.90 |  | (Intercept) | -5.75 | 5.16 | -1.12 | 0.27 |
| Group V6 | 4.61 | 1.36 | 3.39 | **0.00** |  | Group V6 | 4.96 | 1.06 | 4.66 | **0.00** |
| Maternal education2 | -1.11 | 4.29 | -0.26 | 0.80 |  | Maternal education2 | -2.44 | 3.36 | -0.73 | 0.47 |
| Maternal education3 | -1.92 | 4.29 | -0.45 | 0.66 |  | Maternal education3 | 0.29 | 3.35 | 0.09 | 0.93 |
| Maternal age | 0.09 | 0.12 | 0.73 | 0.47 |  | Maternal age | 0.02 | 0.10 | 0.22 | 0.83 |
| Infant gender1 | 0.84 | 1.44 | 0.58 | 0.56 |  | Infant gender1 | -1.60 | 1.13 | -1.42 | 0.17 |
| Infant age at test | -0.82 | 0.59 | -1.38 | 0.18 |  | Infant age at test | 0.88 | 0.46 | 1.90 | 0.07 |
|  | | | | |  |  | | | | |
| **MODEL FIT:** 2 maternal education removed | | | | |  | **MODEL FIT:** 2 maternal education removed | | | | |
| F(4,36) = 6.26, p = 0.00*** | | | | |  | F(4,36) = 6.56, p = 0.00*** | | | | |
| R² = 0.41 | | | | |  | R² = 0.42 | | | | |
| Adj. R² = 0.34 | | | | |  | Adj. R² = 0.36 | | | | |
|  | | | | |  |  | | | | |
| Standard errors: OLS | | | | |  | Standard errors: OLS | | | | |
|  | Est. | S.E. | t | p |  |  | Est. | S.E. | t | p |
| (Intercept) | -3.35 | 4.01 | -0.84 | 0.41 |  | (Intercept) | -9.15 | 3.32 | -2.75 | 0.01 |
| Group V6 | 5.26 | 1.25 | 4.20 | **0.00** |  | Group V6 | 4.77 | 1.04 | 4.60 | **0.00** |
| Maternal age | 0.11 | 0.11 | 1.00 | 0.33 |  | Maternal age | 0.07 | 0.09 | 0.81 | 0.42 |
| Infant gender1 | 0.94 | 1.30 | 0.72 | 0.47 |  | Infant gender1 | -1.55 | 1.08 | -1.44 | 0.16 |
| Infant age at test | -0.73 | 0.54 | -1.34 | 0.19 |  | Infant age at test | 1.09 | 0.45 | 1.43 | 0.12 |
|  | | | | |  |  | | | | |
| **MODEL FIT:** 3 maternal age removed | | | | |  | **MODEL FIT:** 3 maternal age removed | | | | |
| F(5,32) = 3.92, p = 0.01** | | | | |  | F(5,32) = 6.31, p = 0.00*** | | | | |
| R² = 0.38 | | | | |  | R² = 0.50 | | | | |
| Adj. R² = 0.28 | | | | |  | Adj. R² = 0.42 | | | | |
|  | | | | |  |  | | | | |
| Standard errors: OLS | | | | |  | Standard errors: OLS | | | | |
|  | Est. | S.E. | t | p |  |  | Est. | S.E. | t | p |
| (Intercept) | 1.93 | 5.38 | 0.36 | 0.72 |  | (Intercept) | -5.12 | 4.18 | -1.22 | 0.23 |
| Group V6 | 4.56 | 1.35 | 3.38 | **0.00** |  | Group V6 | 4.95 | 1.05 | 4.73 | **0.00** |
| Maternal education2 | -1.46 | 4.23 | -0.35 | 0.73 |  | Maternal education2 | -2.52 | 3.28 | -0.77 | 0.45 |
| Maternal education3 | -2.09 | 4.25 | -0.49 | 0.63 |  | Maternal education3 | 0.25 | 3.30 | 0.08 | 0.94 |
| Infant gender1 | 0.94 | 1.42 | 0.66 | 0.51 |  | Infant gender1 | -1.57 | 1.10 | -1.43 | 0.16 |
| Infant age at test | -0.81 | 0.59 | -1.38 | 0.18 |  | Infant age at test | 0.88 | 0.46 | 1.93 | 0.06 |
| Maternal education: 1 = full primary level; 2 = full secondary level; 3 = college or superior; Infant gender: 1 = female. Est. =estimator; S. E. = standard error; t = t value; p = p value. * = p <0.05; ** = p < 0.01; *** = p value < 0.001. In table S7b The data from the infant which mother reported education level equal to 1 was removed. | | | | | | | | | | |


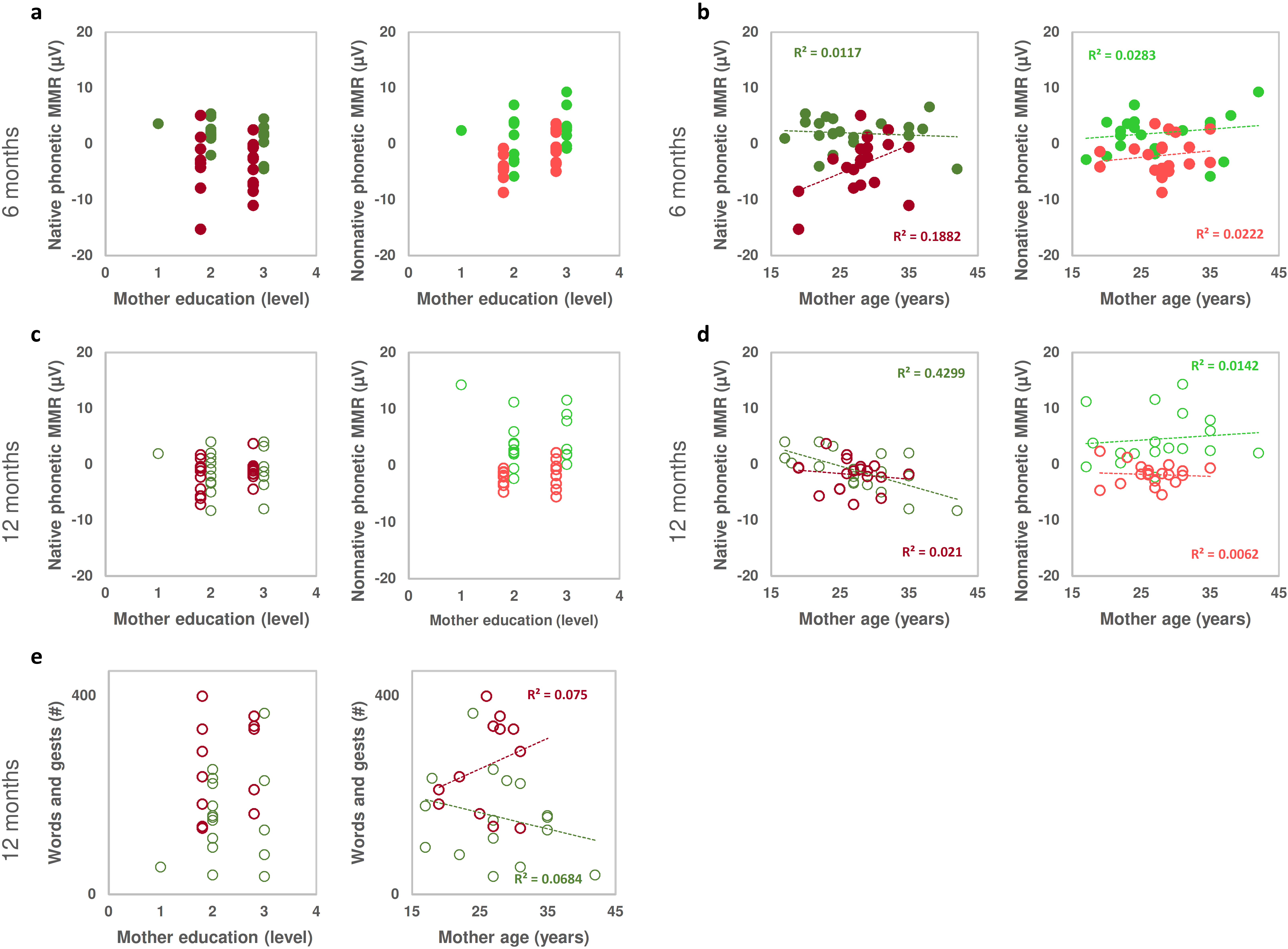


**Figure S3**. The phonetic MMR amplitude at 6 and 12 months of age, and the linguistic abilities at 12 months of age did not significantly associate with maternal education or maternal age, in hRSV and control groups. Clear and dark red draw the data of the infants of the control groups, while clear and dark green depict those from the hRSV groups. We plot, in **(a)** for infants of the groups V6 and C6 and in **(c)** for infants of the groups V12 and C12, the data from of each infant for the native and nonnative phonetic MMR amplitude against the maternal education in levels (1 = full primary, 2 = full secondary and 3 = one or more years of college or professional training). Notice that only one mother reported educational level 1. We depict, in **(b)** for infants of the groups V6 and C6 and in **(d)** for infants of the groups V12 and C12, the bi-variate correlations between the native and nonnative phonetic MMR amplitude against maternal age in years, indicating the R2 for each correlation. In **(e)** we plot the data from the CDI score of each infant against maternal education and maternal age in 12-month-old infants.

| **Table S7a.** Regression models for nonnative phonetic contrast in 12-months-old infants. | | | | |  | **Table S7b.** Regression models for nonnative phonetic contrast in 12-months-old infants, when maternal education is 2 or 3 level only. | | | | |
| --- | --- | --- | --- | --- | --- | --- | --- | --- | --- | --- |
| **MODEL INFO:** | | | | |  | **MODEL INFO:** | | | | |
| Observations: 37 (6 missing obs. deleted) | | | | |  | Observations: 36 | | | | |
| Dependent Variable: Nonnative phonetic MMR amplitude | | | | |  | Dependent Variable: Nonnative phonetic MMR amplitude | | | | |
| Type: OLS linear regression | | | | |  | Type: OLS linear regression | | | | |
|  | | | | |  |  | | | | |
| **MODEL FIT: 1** | | | | |  | **MODEL FIT: 1** | | | | |
| F(6,30) = 8.55, p = 0.00*** | | | | |  | F(5,30) = 6.67, p = 0.00*** | | | | |
| R² = 0.63 | | | | |  | R² = 0.53 | | | | |
| Adj. R² = 0.56 | | | | |  | Adj. R² = 0.45 | | | | |
|  | | | | |  |  | | | | |
| Standard errors: OLS | | | | |  | Standard errors: OLS | | | | |
|  | Est. | S.E. | t | p |  |  | Est. | S.E. | t | p |
| (Intercept) | 15.00 | 9.28 | 1.62 | 0.12 |  | (Intercept) | 4.94 | 8.94 | 0.55 | 0.58 |
| Group V12 | 6.20 | 1.10 | 5.64 | **0.00** |  | Group V12 | 6.20 | 1.10 | 5.64 | **0.00** |
| Maternal age | 0.04 | 0.10 | 0.45 | 0.66 |  | Maternal age | 0.04 | 0.10 | 0.45 | 0.66 |
| Maternal education2 | -10.05 | 3.41 | -2.95 | **0.01** |  | Maternal education3 | 1.35 | 1.08 | 1.26 | 0.22 |
| Maternal education3 | -8.70 | 3.43 | -2.54 | **0.02** |  | Infant gender1 | 0.43 | 1.11 | 0.39 | 0.70 |
| Infant gender1 | 0.43 | 1.11 | 0.39 | 0.70 |  | Infant age at test | -0.68 | 0.73 | -0.94 | 0.36 |
| Infant age at test | -0.68 | 0.73 | -0.94 | 0.36 |  |  | | | | |
|  | | | | |  |  | | | | |
| **MODEL FIT:** 2 maternal education removed | | | | |  | **MODEL FIT:** 2 maternal education removed | | | | |
| F(4,32) = 8.48, p = 0.00*** | | | | |  | F(4,31) = 7.80, p = 0.00*** | | | | |
| R² = 0.51 | | | | |  | R² = 0.50 | | | | |
| Adj. R² = 0.45 | | | | |  | Adj. R² = 0.44 | | | | |
|  | | | | |  |  | | | | |
| Standard errors: OLS | | | | |  | Standard errors: OLS | | | | |
|  | Est. | S.E. | t | p |  |  | Est. | S.E. | t | p |
| (Intercept) | 6.28 | 9.80 | 0.64 | 0.53 |  | (Intercept) | 3.58 | 8.96 | 0.40 | 0.69 |
| Group V12 | 6.76 | 1.19 | 5.70 | **0.00** |  | Group V12 | 6.08 | 1.10 | 5.50 | **0.00** |
| Maternal age | 0.08 | 0.11 | 0.77 | 0.45 |  | Maternal age | 0.04 | 0.10 | 0.40 | 0.70 |
| Infant gender1 | 1.36 | 1.18 | 1.15 | 0.26 |  | Infant gender1 | 0.61 | 1.11 | 0.55 | 0.59 |
| Infant age at test | -0.85 | 0.79 | -1.08 | 0.29 |  | Infant age at test | -0.53 | 0.72 | -0.73 | 0.47 |
|  | | | | |  |  | | | | |
| **MODEL FIT:** 3 maternal age removed | | | | |  | **MODEL FIT:** 3 maternal age removed | | | | |
| F(5,31) = 10.49, p = 0.00*** | | | | |  | F(4,31) = 8.51, p = 0.00*** | | | | |
| R² = 0.63 | | | | |  | R² = 0.52 | | | | |
| Adj. R² = 0.57 | | | | |  | Adj. R² = 0.46 | | | | |
|  | | | | |  |  | | | | |
| Standard errors: OLS | | | | |  | Standard errors: OLS | | | | |
|  | Est. | S.E. | t | p |  |  | Est. | S.E. | t | p |
| (Intercept) | 15.24 | 9.14 | 1.67 | 0.11 |  | (Intercept) | 4.96 | 8.83 | 0.56 | 0.58 |
| Group V12 | 6.19 | 1.08 | 5.71 | **0.00** |  | Group V12 | 6.19 | 1.08 | 5.71 | **0.00** |
| Maternal education2 | -10.28 | 3.32 | -3.09 | **0.00** |  | Maternal education3 | 1.34 | 1.06 | 1.26 | 0.22 |
| Maternal education3 | -8.95 | 3.34 | -2.68 | **0.01** |  | Infant gender1 | 0.39 | 1.09 | 0.36 | 0.72 |
| Infant gender1 | 0.39 | 1.09 | 0.36 | 0.72 |  | Infant age at test | -0.59 | 0.69 | -0.86 | 0.40 |
| Infant age at test | -0.59 | 0.69 | -0.86 | 0.40 |  |  | | | | |
| In table S7b we removed the unique case reporting maternal educational level equal to 1. Maternal education: 1 = full primary level; 2 = full secondary level; 3 = college or superior; Infant gender: 1 = female. Est. =estimator; S. E. = standard error; t = t value; p = p value. * = p <0.05; ** = p < 0.01; *** = p value < 0.001 | | | | | | | | | | |

| **Table S8a.** Regression models for CDI score in 12-months-old infants. | | | | |  | **Table S8b.** Regression models for CDI score in 12-months-old infants, when maternal education levels are 2 or 3 only. | | | | |
| --- | --- | --- | --- | --- | --- | --- | --- | --- | --- | --- |
| **MODEL INFO:** | | | | |  | **MODEL INFO:** | | | | |
| Observations: 29 (14 missing obs. deleted) | | | | |  | Observations: 28 (15 missing obs. deleted) | | | | |
| Dependent Variable: CDI | | | | |  | Dependent Variable: CDI | | | | |
| Type: OLS linear regression | | | | |  | Type: OLS linear regression | | | | |
|  | | | | |  |  | | | | |
| **MODEL FIT: 1** | | | | |  | **MODEL FIT: 1** | | | | |
| F(6,22) = 1.79, p = 0.15 | | | | |  | F(5,21) = 1.67, p = 0.19 | | | | |
| R² = 0.34 | | | | |  | R² = 0.28 | | | | |
| Adj. R² = 0.15 | | | | |  | Adj. R² = 0.11 | | | | |
|  | | | | |  |  | | | | |
| Standard errors: OLS | | | | |  | Standard errors: OLS | | | | |
|  | Est. | S.E. | t | p |  |  | Est. | S.E. | t | p |
| (Intercept) | 342.33 | 299.42 | 1.14 | 0.27 |  | (Intercept) | 472.84 | 292.16 | 1.62 | 0.12 |
| Group V12 | -88.02 | 38.36 | -2.29 | **0.03** |  | Group V12 | -88.02 | 38.36 | -2.29 | **0.03** |
| Maternal age | -0.02 | 3.27 | -0.01 | 0.99 |  | Maternal age | -0.02 | 3.27 | -0.01 | 0.99 |
| Maternal education2 | 130.52 | 105.64 | 1.24 | 0.23 |  | Maternal education3 | 25.01 | 39.44 | 0.63 | 0.53 |
| Maternal education3 | 155.53 | 108.73 | 1.43 | 0.17 |  | Infant gender1 | 31.10 | 39.83 | 0.78 | 0.44 |
| Infant gender1 | 31.10 | 39.83 | 0.78 | 0.44 |  | Infant age at test | -18.09 | 23.67 | -0.76 | 0.45 |
| Infant age at test | -18.09 | 23.67 | -0.76 | 0.45 |  |  | | | | |
|  | | | | |  |  | | | | |
| **MODEL FIT:** 2 maternal education removed | | | | |  | **MODEL FIT:** 2 maternal education removed | | | | |
| F(4,23) = 2.14, p = 0.11 | | | | |  | F(4,22) = 2.04, p = 0.12 | | | | |
| R² = 0.27 | | | | |  | R² = 0.27 | | | | |
| Adj. R² = 0.14 | | | | |  | Adj. R² = 0.14 | | | | |
|  | | | | |  |  | | | | |
| Standard errors: OLS | | | | |  | Standard errors: OLS | | | | |
|  | Est. | S.E. | t | p |  |  | Est. | S.E. | t | p |
| (Intercept) | 394.19 | 286.76 | 1.37 | 0.18 |  | (Intercept) | 443.55 | 284.53 | 1.56 | 0.13 |
| Group V12 | -99.79 | 37.56 | -2.66 | **0.01** |  | Group V12 | -91.14 | 37.52 | -2.43 | **0.02** |
| Maternal age | -0.95 | 3.21 | -0.30 | 0.77 |  | Maternal age | -0.17 | 3.22 | -0.05 | 0.96 |
| Infant gender1 | 18.20 | 38.14 | 0.48 | 0.64 |  | Infant gender1 | 33.09 | 39.16 | 0.85 | 0.41 |
| Infant age at test | -8.99 | 22.72 | -0.40 | 0.70 |  | Infant age at test | -14.76 | 22.77 | -0.65 | 0.52 |
|  | | | | |  |  | | | | |
| **MODEL FIT:** 3 maternal age removed | | | | |  | **MODEL FIT:** 3 maternal age removed | | | | |
| F(5,22) = 2.25, p = 0.09 | | | | |  | F(4,22) = 2.19, p = 0.10 | | | | |
| R² = 0.34 | | | | |  | R² = 0.28 | | | | |
| Adj. R² = 0.19 | | | | |  | Adj. R² = 0.15 | | | | |
|  | | | | |  |  | | | | |
| Standard errors: OLS | | | | |  | Standard errors: OLS | | | | |
|  | Est. | S.E. | t | p |  |  | Est. | S.E. | t | p |
| (Intercept) | 342.16 | 291.55 | 1.17 | 0.25 |  | (Intercept) | 472.80 | 285.37 | 1.66 | 0.11 |
| Group V12 | -88.03 | 37.42 | -2.35 | **0.03** |  | Group V12 | -88.03 | 37.42 | -2.35 | **0.03** |
| Maternal education2 | 130.64 | 101.66 | 1.29 | 0.21 |  | Maternal education3 | 25.03 | 38.44 | 0.65 | 0.52 |
| Maternal education3 | 155.67 | 104.21 | 1.49 | 0.15 |  | Infant gender1 | 31.14 | 38.45 | 0.81 | 0.43 |
| Infant gender1 | 31.14 | 38.45 | 0.81 | 0.43 |  | Infant age at test | -18.13 | 22.28 | -0.81 | 0.42 |
| Infant age at test | -18.13 | 22.28 | -0.81 | 0.42 |  |  | | | | |
| In table S8b we removed the unique case reporting education level equal to 1. Maternal education: 1 = full primary level; 2 = full secondary level; 3 = college or superior; Infant gender: 1 = female. Est. =estimator; S. E. = standard error; t = t value; p = p value. * = p <0.05; ** = p < 0.01; *** = p value < 0.001. | | | | | | | | | | |

| **Table S9**. Regression models for native and nonnative phonetic MMR, including "severity" of the desease as a factor, in group V6. | | | | | | | | | | |
| --- | --- | --- | --- | --- | --- | --- | --- | --- | --- | --- |
| **MODEL INFO:** | | | | |  | **MODEL INFO:** | | | | |
| Observations: 19 (6 missing obs. deleted) | | | | |  | Observations: 19 (6 missing obs. deleted) | | | | |
| Dependent Variable: Native phonetic MMR amplitude | | | | |  | Dependent Variable: Nonnative phonetic MMR amplitude | | | | |
| Type: OLS linear regression | | | | |  | Type: OLS linear regression | | | | |
|  | | | | |  |  | | | | |
| **MODEL FIT:** | | | | |  | **MODEL FIT:** | | | | |
| F(6,12) = 0.81, p = 0.58 | | | | |  | F(6,12) = 1.75, p = 0.19 | | | | |
| R² = 0.29 | | | | |  | R² = 0.47 | | | | |
| Adj. R² = -0.07 | | | | |  | Adj. R² = 0.20 | | | | |
|  | | | | |  |  | | | | |
| Standard errors: OLS | | | | |  | Standard errors: OLS | | | | |
|  | Est. | S.E. | t | p |  |  | Est. | S.E. | t | P |
| (Intercept) | 1.58 | 6.48 | 0.24 | 0.81 |  | (Intercept) | -2.65 | 8.07 | -0.33 | 0.75 |
| Severity | 0.47 | 0.41 | 1.13 | 0.28 |  | Severity | -0.01 | 0.51 | -0.02 | 0.98 |
| Maternal age | -0.06 | 0.10 | -0.53 | 0.60 |  | Maternal age | -0.08 | 0.13 | -0.63 | 0.54 |
| Maternal education2 | -0.48 | 3.13 | -0.15 | 0.88 |  | Maternal education2 | -2.04 | 3.90 | -0.52 | 0.61 |
| Maternal education3 | -1.41 | 3.20 | -0.44 | 0.67 |  | Maternal education3 | -1.44 | 3.98 | -0.36 | 0.72 |
| Infant gender1 | 0.50 | 1.37 | 0.37 | 0.72 |  | Infant gender1 | -2.01 | 1.70 | -1.18 | 0.26 |
| Infant age at test | -0.41 | 0.64 | -0.64 | 0.54 |  | Infant age at test | 2.07 | 0.80 | 1.59 | 0.32 |
|  | | | | |  |  | | | | |
|  | | | | | | | | | | |
| **Table S10**. Regression models for nonnative phonetic MMR and CDI, including "severity" of the desease as a factor, in group V12. | | | | | | | | | | |
| **MODEL INFO:** | | | | |  | **MODEL INFO:** | | | | |
| Observations: 18 (2 missing obs. deleted) | | | | |  | Observations: 16 (4 missing obs. deleted) | | | | |
| Dependent Variable: Nonnative phonetic MMR amplitude | | | | |  | Dependent Variable: CDI | | | | |
| Type: OLS linear regression | | | | |  | Type: OLS linear regression | | | | |
|  | | | | |  |  | | | | |
| **MODEL FIT:** | | | | |  | **MODEL FIT:** | | | | |
| F(6,11) = 1.19, p = 0.38 | | | | |  | F(6,9) = 0.26, p = 0.94 | | | | |
| R² = 0.39 | | | | |  | R² = 0.15 | | | | |
| Adj. R² = 0.06 | | | | |  | Adj. R² = -0.42 | | | | |
|  | | | | |  |  | | | | |
| Standard errors: OLS | | | | |  | Standard errors: OLS | | | | |
|  | Est. | S.E. | t | p |  |  | Est. | S.E. | t | p |
| (Intercept) | 23.31 | 17.80 | 1.31 | 0.22 |  | (Intercept) | 127.28 | 452.45 | 0.28 | 0.78 |
| Severity | 0.32 | 0.61 | 0.52 | 0.62 |  | Severity | -2.57 | 17.38 | -0.15 | 0.89 |
| Maternal age | 0.13 | 0.19 | 0.71 | 0.49 |  | Maternal age | -2.96 | 4.65 | -0.64 | 0.54 |
| Maternal education2 | -8.14 | 5.69 | -1.43 | 0.18 |  | Maternal education2 | 90.37 | 141.58 | 0.64 | 0.54 |
| Maternal education3 | -6.49 | 5.46 | -1.19 | 0.26 |  | Maternal education3 | 96.30 | 135.96 | 0.71 | 0.50 |
| Infant gender1 | 1.21 | 2.52 | 0.48 | 0.64 |  | Infant gender1 | 5.44 | 66.33 | 0.08 | 0.94 |
| Infant age at test | -1.41 | 1.54 | -0.91 | 0.38 |  | Infant age at test | 3.32 | 37.57 | 0.09 | 0.93 |
|  | | | | |  |  | | | | |
